# Parenting Styles and Internalizing Symptoms in Adolescence: A Systematic Literature Review

**DOI:** 10.3390/ijerph16173192

**Published:** 2019-09-01

**Authors:** Arantxa Gorostiaga, Jone Aliri, Nekane Balluerka, Joanes Lameirinhas

**Affiliations:** Faculty of Psychology, University of the Basque Country UPV/EHU, 20018 Donostia, Spain

**Keywords:** systematic review, parenting practices and styles, depression, anxiety, suicidal ideation, adolescents

## Abstract

A growing body of recent research has identified associations between various parenting practices and styles and internalizing problems among adolescents. However, the reported findings are inconsistent and the studies in question have been conducted from different theoretical backgrounds. The aim of this systematic review was to synthesize the literature on the association of parental socialization styles with depression, anxiety, and suicidal ideation. To this end, we conducted a systematic search of the PsycInfo, Scopus, Pubmed, and Web of Science databases, covering literature published from 2010 to 2019. The search was restricted to peer-reviewed studies in English or Spanish. The results show that parental warmth, behavioural control, and autonomy granting are inversely related to internalizing symptoms in adolescents. Conversely, psychological control and harsh control by parents are positively associated with adolescent anxiety, depression, and suicidal ideation. Although the associated effect sizes are only small or moderate, the results suggest that these variables should be taken into account when designing programmes aimed at promoting parenting styles conducive to the wellbeing of adolescents.

## 1. Introduction

There is a growing interest in the study of child rearing, largely due to the development of theoretical models such as attachment theory [1,2,3] and social learning theory [4], which emphasize the influence that parents have on children’s development. Research on child rearing has particularly focused on the role played by general patterns of parental behaviour, referred to as parenting styles. Darling and Steinberg [5] consider parenting style to be a constellation of attitudes, goals, and patterns of child rearing that shape the emotional climate of the parent-child relationship and which remain constant across different life situations.

Building on the work of Baumrind [6] and her description of authoritative, authoritarian, and permissive parenting, Maccoby and Martin [7] proposed a model of parenting styles based on two broad orthogonal dimensions, demandingness and responsiveness, thus giving rise to a typology involving four potential parenting styles: authoritative or democratic (high demandingness and high responsiveness), authoritarian (high demandingness and low responsiveness), indulgent (low demandingness and high responsiveness), and uninvolved or neglectful (low demandingness and low responsiveness). According to Maccoby and Martin [7], the first of these dimensions refers to the limits and rules which parents set in order to regulate their child’s behaviour, and to the demand that the child complies with them. The second dimension concerns the extent to which parents show affection, are involved with and accepting of their child’s behaviour and feelings, and are sensitive to his or her needs. It must be noted that other theoretical approaches to parenting styles have highlighted the abovementioned orthogonal dimensions, also called acceptance/involvement and strictness/imposition [8,9].

While parenting styles refer to general patterns of parental behaviour, parental practices constitute more specific forms of parent-child interaction in specific situations [5]. For example, behavioural control refers to the ways in which parents monitor or supervise their child’s behaviour to ensure that it is appropriate [10,11]. The limits and rules which parents set in this respect can have a positive impact on the child’s psychosocial adjustment. However, harsh control, defined as physical and/or verbal punishment by parents, may undermine the child’s adjustment [12]. As for psychological control, this refers to parents’ attempts to manipulate their child’s thoughts and feelings by inducing a sense of guilt or humiliation, or through emotional blackmail or overprotectiveness [13,14]. Another theme that has been studied within the framework of child rearing practices is autonomy granting, defined by McLeod et al. [15] as “parental encouragement of children’s opinions and choices, acknowledgement of children’s independent perspectives on issues, and solicitation of children’s input on decisions and solutions of problems” (p. 162). Finally, parental warmth is described as “the sense of positive regard expressed by the parent toward the adolescent, pleasant interactions shared between parent and adolescent, or parental involvement in the adolescent’s activities” [16] (p. 12).

Various authors have stated that, as parenting styles, parental practices can also be classified by the acceptance/involvement and strictness/imposition dimensions [17,18,19,20]. Therefore, behavioural control is characterized by a high acceptance/involvement and strictness/imposition, which fits with authoritative style. However, harsh control and psychological control are characterized by a low acceptance and a high strictness/imposition, related to authoritarian style [21]. Likewise, autonomy granting is defined by a high acceptance/involvement and low strictness/imposition, which are associated with the indulgent style [22]. Finally, parental warmth is related to a high acceptance/involvement, but it remains unclear whether this parental practice is characteristic of authoritative or indulgent style [19,22].

These theoretical models have formed the basis for numerous studies examining the role played by different parenting practices in relation to the psychological adjustment of children and adolescents. Therefore, for instance, various meta-analyses have found a negative correlation between parental warmth and internalizing symptoms such as anxiety [15,16,23,24,25] and depression in young people [26,27]. As regards to harsh control and psychological control, both have been associated with more internalizing symptoms in children and adolescents [28], whereas behavioural control has been linked to fewer symptoms of this kind [12,29,30]. With respect to autonomy granting, several meta-analyses have found a negative correlation between this practice and children’s internalizing symptoms [12,15,16]. In addition, some studies have reported an association between suicidal ideation and psychological control [31], as well as an inverse relationship between this kind of control and both maternal warmth [32,33,34] and autonomy granting [33], although no meta-analyses have so far examined these relationships.

With respect to the different types of parenting styles, various meta-analyses have concluded that authoritarian and neglectful parenting are associated with the presence of internalizing symptoms in children [12,23,26], probably due to the lack of acceptance/involvement which characterizes these parenting styles. It should be noted, however, that not all analyses have found a statistically significant correlation in this respect (e.g., [24]). As regards authoritative parenting, this has been associated with fewer internalizing symptoms and, in general, better psychological adjustment in children and adolescents [12,26,35]. Studies which have examined the relationship between parenting styles and suicide risk in young people have found that authoritarian parenting is associated with an increased risk of suicide [36,37,38], whereas authoritative parenting appears to be related to a lower risk [36,37].

Although different theoretical models consider that children’s upbringing has an important impact on their subsequent psychological adjustment, several studies have found only a moderate effect size for this relationship [15,27,39]. This could be due to an overly simplistic conception of the relationship between child rearing and psychopathology, since there are multiple causes of the latter [12]. Consequently, these studies highlight the need to consider other variables which may moderate the relationship between parenting styles and internalizing symptoms, for example, genetic factors [15,27,39], parental gender [16,23,24,39], anxiety or depression in the father or mother [40], and the child’s age and gender [15,23,27,39]. Furthermore, and as Darling and Steinberg [5] point out, parenting style itself is more likely to be a moderator than a predictor of children’s psychosocial outcomes. It should also be borne in mind that the abovementioned studies have been conducted in western countries with participants of a medium socioeconomic status, factors which may also moderate the relationship between child rearing and internalizing symptoms [23].

Studies that have examined the relationship between parenting styles and internalizing symptoms in children and adolescents also suffer from a number of methodological limitations, such as the almost exclusive reliance on questionnaires, some of which lack convergent validity, and this highlights the importance of employing other assessment techniques [15,23,25]. In addition, the fact that the majority of studies are based on non-experimental cross-sectional designs makes it difficult to establish causal hypotheses, and hence there is a need for experimental and/or longitudinal studies in this field [25].

A further issue to consider is the lack of consensus in conceptualizing the different parenting style dimensions [12,25]. More specifically, although there is agreement with respect to the definition of the acceptance/involvement dimension, this is not the case for strictness/imposition [29]. A more precise definition of these dimensions is therefore necessary so that future studies may be based on a consistent set of criteria and constructs [23].

In light of the above, the goal of this systematic review is to synthesize the literature on the association of parental socialization styles and practices with anxiety, depression, and suicidal ideation in youth. In doing so, we aim to fill the gap in the literature regarding the relationship between different parenting style dimensions and practices and suicidal ideation in adolescents, while also examining the methods and characteristics of studies carried out over the past decade on the relationship between parenting styles and practices and depression, anxiety, and suicidal ideation.

## 2. Method

This systematic review was conducted in accordance with PRISMA (Preferred Reporting Items for Systematic reviews and Meta-Analyses) statement guidelines [41].

### 2.1. Search Strategy

Systematic searches of the PsycInfo, Scopus, Pubmed, and Web of Science databases were conducted on 15 March 2019, covering literature published from 2010 to 2019. The search terms included ‘parenting styles’, ‘parenting practices’, ‘parental socialization’, ‘anxiety’, ‘anxious’, ‘depressive’, ‘depression’, and ‘suicid*’. The asterisk (*) acts as a truncation symbol to detect various results composed of a single string of text: for example, ‘suicid*’ detects terms such as ‘suicide’ and ‘suicidal’. Full search strategies are presented in Table 1, Table 2, Table 3 and Table 4.

### 2.2. Study Selection

Two researchers (A.G. and J.A.) conducted the first screening of articles based on titles and abstracts. Inter-rater reliability was satisfactory, with a kappa coefficient of 0.93. Any disagreement was resolved by consensus. Eligible articles were then identified by screening full texts using previously agreed exclusion and inclusion criteria (see below). In this case, the kappa coefficient was 0.81, and any disagreements were once again resolved through consensus.

### 2.3. Eligibility Criteria

The inclusion criteria were (1) studies that assess anxiety, depression, and/or suicidal ideation in adolescents aged between 12 and 18 years, and which examine parenting styles or practices; (2) articles published in English or Spanish; and (3) studies that analyse the relationship between parenting styles or practices and anxiety, depression, and/or suicidal ideation in adolescents. 

**Figure 1:**
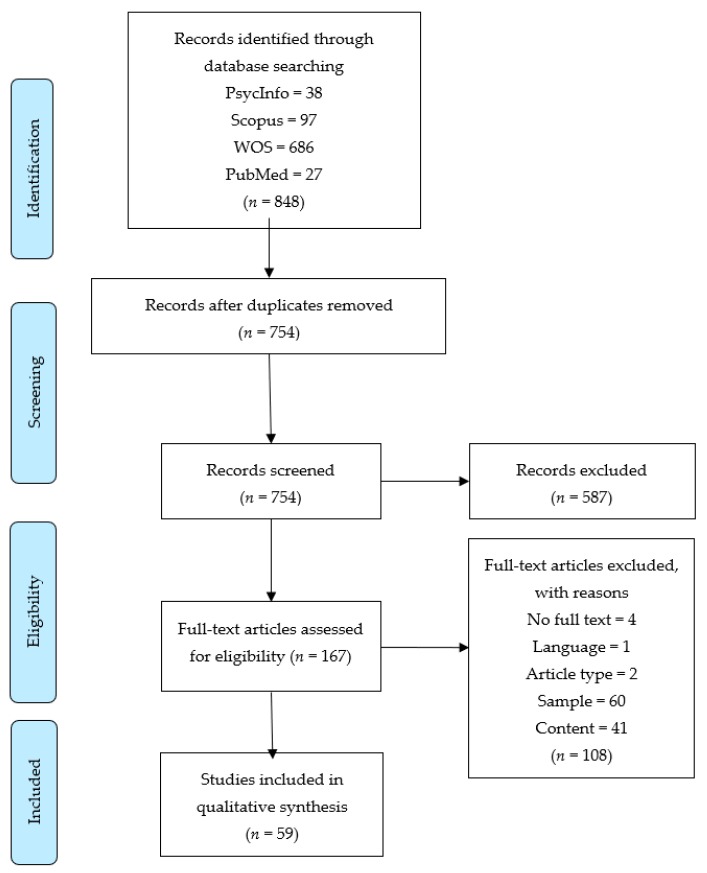
Preferred Reporting Items for Systematic reviews and Meta-Analyses (PRISMA) flow diagram.

Accordingly, the exclusion criteria were (1) not having access to the full text (no full text); (2) publications in languages other than English or Spanish (language); (3) theoretical reviews, meta-analyses, systematic reviews, or studies in which no data were collected (article type); (4) studies whose participants were not adolescents aged between 12 and 18 years (sample); and (5) studies that did not examine the relationship between parenting styles or practices and anxiety, depression, and/or suicidal ideation in adolescents (content). As more than one of these exclusion criteria may apply to the same study, records were coded following the order in which the criteria are presented here (see Figure 1).

### 2.4. Data Extraction

All studies were coded using a detailed coding manual and the following data were extracted (where available) from each: country of study, study type/design, sample size/description, age of participants, gender, parenting assessment, depression assessment, anxiety assessment, suicidal ideation assessment, and main results.

In order to evaluate parenting practices, we considered the following practices: parental warmth, which refers to measures of parental care, acceptance, involvement, emotional support, concern, and understanding; psychological control, referring to parental practices that manipulate children’s psychological experiences through imposition or conditional love; behavioural control, which refers to parental monitoring or supervision; harsh control, that is, the use of corporal punishment or severe punishment practices; and autonomy granting, referring to practices that explicitly foster children’s autonomy. We also considered other practices, such as inconsistent discipline, negative parenting, or over-reactivity, which appear less frequently in the reviewed literature. Finally, we coded the four parenting styles (authoritative, authoritarian, indulgent, and neglectful).

Regarding the outcome variables, it should be noted that suicidal ideation was considered in a broad sense. Therefore, our conceptualization included the lifetime prevalence of suicide attempts, suicidal thoughts, self-harm, or self-destructive thoughts and behaviours.

## 3. Results

### 3.1. Literature Search Results

The search strategy yielded 848 results, as shown in Figure 1. After removing duplicates, a first screening based on title and abstracts resulted in 167 articles. Finally, after a full-text read-through, 59 studies [31,36,42,43,44,45,46,47,48,49,50,51,52,53,54,55,56,57,58,59,60,61,62,63,64,65,66,67,68,69,70,71,72,73,74,75,76,77,78,79,80,81,82,83,84,85,86,87,88,89,90,91,92,93,94,95,96,97,98] were selected for inclusion (see Table 5).

### 3.2. Study Characteristics

Half of the included studies (N = 29; see Table 5) had been published since 2016. Overall, the studies reviewed had been conducted in 28 different countries, most notably the USA (33.3% of studies), followed by China (11.7%), and Mexico, Portugal, and Spain (each 5%). A cross-sectional design was used in 78% of studies, while the remaining 22% were longitudinal. Sample size ranged from 27 [65] to 71,891 [36], with a median of 445 participants. The proportion of females in the samples ranged between 0% [89] and 92.7% [88], with a median of 52%.

Regarding outcome variables, 66.1% of studies assessed depression, 27.1% anxiety, and 18.6% suicidal ideation. In approximately 15% of studies, the results for boys and girls were analysed separately, while in 34%, the results relating to parenting style were analysed separately for mothers and fathers.

Parenting practices were assessed by several different instruments. Specifically, warmth was most often assessed with the EMBU (Egna Minnen Beträffande Uppfostran), an inventory for assessing memories of parental rearing behaviour ([99]; 7 studies); the Alabama Parenting Questionnaire (APQ; [100,101]; 5 studies); the Child’s Report of Parental Behaviour Inventory (CRPBI; [102]; 4 studies); the Andrade and Betancourt Parental Practices Scale (Andrade and Betancourt PPS; [103]; 3 studies); the Parenting Bonding Instrument (PBI; [104]; 2 studies); the Parental Socialization Scale (PSS; [29]; 2 studies); and related measures (21 studies). Psychological control was most often assessed with the EMBU (7 studies), the CRPBI (3 studies), the Andrade and Betancourt PPS (3 studies), the PSS (2 studies), the Psychological Control Scale (PCS; [13]; 2 studies), the Parent Behaviour Measure (PBM; [105]; 2 studies), and related measures (11 studies). Behavioural control was most often assessed with the APQ (5 studies), the CRPBI (4 studies), the Andrade and Betancourt PPS (3 studies), the PSS (2 studies), the PBM (2 studies), and related measures (15 studies). Harsh control was most often assessed with the APQ (5 studies), the PBM (2 studies), and related measures (9 studies). Autonomy granting was most often assessed with the Andrade and Betancourt PPS (3 studies), the PSS (2 studies), and related measures (3 studies). Finally, parenting styles were assessed either with measures created ad hoc (5 studies), or with instruments such as the Parenting Practices Scale [106].

In addition to parenting, this review is also concerned with depression, anxiety, and suicidal tendencies in adolescents. Depression was most often assessed with the Clinical Diagnostic Interview (CDI; [107]; 10 studies), the Center for Epidemiologic Studies Depression Scale (CES-D; [108]; 10 studies), the Youth Self Report (YSR; [109]; 5 studies), the Symptom Checklist 90 Revised (SCL-90-R; [110]; 3 studies), the Diagnostic Interview Schedule for Children (DISC; [111]; 2 studies), the Beck Depression Inventory (BDI; [112]; 2 studies), and related measures (7 studies). Anxiety was most often measured with the Revised Child Anxiety and Depression Scale (RCADS; [113]; 2 studies), the Revised Children’s Manifest Anxiety Scale (RCMAS; [114]; 2 studies), and related measures (12 studies). Finally, suicidal ideation was most often assessed either with measures created ad hoc (6 studies), or with selected items from standardized measurement instruments designed to assess other constructs (2 studies).

### 3.3. Association of Parenting Practices and Styles with Depression, Anxiety, and Suicidal Ideation in Adolescents

The results of our analysis showed an inverse relationship of moderate effect size between parental warmth and depressive symptoms in adolescents. Correlation coefficients ranged between −0.75, reported by Jelenova et al. [65] in a sample of adolescents with inflammatory bowel disease, and −0.03, with a median of −0.26. Regarding the inverse association between parental warmth and anxiety, the median correlation indicated a small effect size (−0.14), with coefficients ranging between −0.51 (once again, in the study by Jelenova et al. [65]) and a minimum of −0.14. The relationship between parental warmth and suicidal ideation also showed a small effect size (median correlation of −0.17), with coefficients ranging between −0.71 ([50], in a sample of adolescents who had assaulted their parents) and 0.03.

In general, there was a positive relationship with a small or moderate effect size (median 0.24) between parental psychological control and depression in adolescents, with correlation coefficients ranging between 0.48 [42] and 0.05. The effect size for the relationship between psychological control and anxiety was smaller (median 0.16), and in this case, coefficients ranged between 0.47 [78] and 0.03. Finally, the relationship between psychological control and suicidal ideation yielded a median correlation of 0.20, ranging between 0.41 [83] and −0.08.

Behavioural control showed an inverse association of a small effect size with depression in adolescents (median −0.02), with coefficients ranging between −0.53 ([88], in a sample of adolescents hospitalized for a suicide attempt) and −0.03. This parenting practice was only weakly associated with anxiety (median −0.03), with coefficients ranging between −0.19 [85] and 0.07. Finally, the relationship between behavioural control and suicidal ideation was almost null, with the highest coefficient being −0.12, in the study by Florenzano et al. [61].

Harsh control showed a positive association of a moderate effect size (median 0.25) with depression in adolescents, with coefficients ranging between 0.78 [65] and 0.14. The effect size was also moderate (median 0.34) for the relationship between this practice and anxiety, and in this case, coefficients ranged between 0.68 [65] and 0.24. An association of a moderate effect size was also found for the relationship between harsh control and suicidal ideation, although this is based on just two studies; the highest correlation was 0.28, in the study by Nunes and Mota [80].

Finally, autonomy granting showed a negative association of a moderate effect size with depression in adolescents (median −0.28), and a slightly weaker inverse relationship with anxiety (median −0.22) and suicidal ideation (median −0.22). Coefficients ranged between −0.44 [65] and −0.03 for the relationship between autonomy granting and depression, between −0.45 [65] and −0.08 for the association with anxiety, and between −0.35 [83] and −0.02 for the relationship with suicidal ideation.

Regarding parenting styles, indulgent parenting has been found to be associated with less suicidal ideation in adolescents [36], while authoritative parenting has been related to less depression [66,74,86,90]. Conversely, authoritarian [71,74] and negligent parenting styles [71,86] have been associated with more symptoms of depression in adolescents.

With respect to other parenting practices that have been examined in a smaller number of studies, positive associations of a moderate effect size have been reported between adolescent depression and inconsistent discipline [44], negative parenting [46], family dysfunction [49], a desultory parental style [65], and over-reactivity [91]. Conversely, a negative relationship has been observed between adolescent depression and effective parenting [76]. As regards anxiety in young people, a positive relationship has been found with respect to both family dysfunction [49] and overprotection [78]. Finally, one study reported a negative and moderate correlation between suicidal ideation in adolescents and parental imposition [31], while another found a positive association between internalizing behaviour in adolescent girls and parental over-involvement [77].

## 4. Discussion

The main goal of this study was to synthesize the literature on the association of parenting practices and styles with anxiety, depression, and suicidal ideation in adolescents. Specifically, we reviewed 59 studies that analyse the relationship between parenting and one or more outcome variables. The evidence base is largest with respect to depression, followed by anxiety, but there is considerably less research on the relationship between parenting and suicidal ideation in young people.

The fact that the reviewed studies were conducted in almost thirty different countries, with half of them being published since 2016, indicates that the relationship between parenting and internalizing problems in young people continues to be a topic of research interest. Despite this interest, however, some of the problems identified in previous meta-analyses have yet to be resolved. For example, and with respect to a shortcoming noted by Wood et al. [25] and Pinquart [12], the present review shows that there is still considerable heterogeneity in the conceptualization of different parenting styles and practices, making it difficult to draw firm conclusions from the results. This heterogeneity is illustrated by the fact that the studies reviewed used around 40 different instruments, with some being standardized, whilst others were developed ad hoc. There was also notable variation in the parenting practices and styles considered, even across studies that used the same measurement instrument. All these aspects hamper the generalization of results.

As regards the operationalization of outcome variables, the 39 studies which examined depression used 12 different instruments, most frequently the Children’s Depression Inventory (CDI) and the Center for Epidemiologic Studies Depression Scale (CES-D). Anxiety was also operationalized in a variety of ways, and only two instruments, the Revised Child Anxiety and Depression Scale (RCADS) and the Revised Children’s Manifest Anxiety Scale (RCMAS), were used in more than one study. With respect to suicidal ideation, it is noteworthy that instruments designed specifically to measure this construct, such as the Suicidal Ideation Questionnaire (SIQ, [115]) or Suicide Intent Scale (SIS, [116]), were rarely employed. Some studies used two items from the Youth Self-Report (YSR, [117]), namely “I deliberately try to hurt or kill myself” and “I think about killing myself”, and one study used a single ad hoc item, “I’ve thought about ways of killing myself”. It should also be noted, in relation to a point made earlier, that we found very few studies analysing the relationship between suicide and parental practices, and hence, in the present review, suicidal ideation was considered in a broad sense (lifetime prevalence of suicide attempts, suicidal thoughts, self-harm, or self-destructive thoughts and behaviours), including both passive and active ideation [118].

Although various meta-analyses have examined the relationship between parenting practices and depression [26,27,35], anxiety [15,23,25,39,40], or both constructs [12,16,24] in children and adolescents, no meta-analysis or systematic review has considered studies published in the period 2017–2019, and none has included the variable suicidal ideation. The latter, however, may be a precursor of suicidal behaviour [119], and it is therefore an important variable in the context of adolescent wellbeing. Although, in the period considered by this review, only a small number of studies have examined the relationship between parenting practices and suicidal ideation in young people, our analysis suggests that suicidal ideation is negatively associated with parental warmth, behavioural control, and autonomy granting, and positively associated with psychological control and harsh control. It should be noted, however, that the corresponding effect sizes are small. Some research also suggests that indulgent parenting may protect against suicidal thoughts, and a relationship has been reported between suicidal ideation and both parental imposition and over-involvement.

Regarding depression and anxiety, overall, our results are consistent with published findings, since previous meta-analyses have likewise reported a negative relationship between parental warmth and internalizing symptoms in children and adolescents [15,16,23,24,25], as well as a positive association of both psychological control and harsh control with the development of depression and anxiety symptoms [12,29,30]. Regarding behavioural control, and in line with previous meta-analyses, our results showed that this practice was negatively associated with both depression and anxiety, although the average effect size is very small. Also consistent with existing meta-analyses, we found a negative relationship between autonomy granting and internalizing symptoms in adolescents [12,15,16]. We also observed a relationship between adolescent depression and a number of other parenting practices, including inconsistent discipline, negative parenting, family dysfunction, a desultory parental style, over-reactivity, and effective parenting. Additionally, we found an association between anxiety and family dysfunction and overprotection. Yap et al. [16] reported similar results in relation to inconsistent discipline and overprotection. Finally, as regards parenting styles, our results suggest that neglectful and authoritarian parenting are associated with higher levels of depression, whereas an authoritative parenting style is related to less depression. These results are partially consistent with the classic studies conducted in the United States (e.g., [8,9]), although there are studies conducted in other cultural contexts in which the indulgent style is the most optimal (e.g., [120,121]).

In general, and in line with the results of previous meta-analyses (see, for example, [12,15,27]), the effect sizes for these associations were either small or moderate. Therefore, these effect sizes are as expected and are considered adequate, since many other potentially important variables are related to internalizing problems in adolescents. However, this does not mean that parental practices are not relevant in terms of generating or protecting against such problems. Therefore, several primary studies have also pointed out the importance of such results, despite the effect sizes found [8,122]. In this respect, it is worth noting that some of the studies included in this systematic review, which report the largest effect sizes, have involved clinical samples of young people, for example, adolescents with inflammatory bowel disease [65], adolescents who have assaulted their parents [50], or adolescents hospitalized following a suicide attempt [88]. This suggests that it is in these kinds of populations where adequate parenting practices may have a greater protective effect in relation to adolescent wellbeing.

This review has certain limitations. First, the considerable variation in the conceptualization of parental socialization and the diverse range of instruments used hampers the comparison of results across studies. A related issue here is that, despite having conducted an exhaustive database search, it is possible that some studies were missed due to their use of different terminology. A further limitation is that although we considered a broad period (2010–2019), the number of studies reviewed was not sufficient to analyse the influence of potential moderator variables, such as sample characteristics, cultural context, participants’ gender, parental gender, research design, or the quality of the measurement instruments used. In this regard, it should be noted that many studies have had to be excluded due to two main reasons: that the participants were not adolescents between the ages of 12 and 18 and that the studies did not examine the relationship of parenting practices or styles with anxiety, depression, and/or suicidal ideation in adolescents. Therefore, for example, it was common to find studies in which anxiety or depression had been evaluated in parents and not in adolescents, or studies in which participants were adults. Finally, our systematic review has been limited to studies that have used questionnaires and this fact may be related to the effect sizes found.

As areas of future research, it is worth mentioning the need to systematically analyse the parental styles that may be more effective for the psychosocial adjustment of adolescents depending on the cultural context, since, as previously noted, there is no consensus on this issue. On the other hand, a systematic review could be carried out with various operationalizations of parental socialization.

## 5. Conclusions

The studies reviewed suggest that parental warmth, behavioural control, and autonomy granting are inversely associated with internalizing problems, especially depression, in adolescents. Conversely, psychological control and harsh control by parents show a positive relationship with adolescent anxiety, depression, and suicidal ideation. Although the associated effect sizes are small or moderate, the results suggest that these variables should be taken into account when designing programmes aimed at promoting parenting styles conducive to the wellbeing of adolescents.

## Figures and Tables

**Table 1:** Search Strategy in PsycInfo.

Search Number	Search Terms	Results
S1	KW parenting styles OR KW parenting practices OR KW parental socialization	639
S2	KW anxiety OR KW anxious	24,447
S3	KW depressive OR KW depression	44,844
S4	KW suicid *	10,868
S5	S1 AND (S2 OR S3 OR S4)	38

Limiters: Published Date: 20100101-20191231; Language: English, Spanish; Document Type: Journal Article Search modes—Boolean/Phrase. Note: The asterisk (*) acts as a truncation symbol to detect various results composed of a single string of text.

**Table 2:** Search Strategy in Scopus.

Search Number	Search Terms	Results
S1	(KEY (“parenting styles”) OR KEY (“parenting practices”) OR KEY (“parental socialization”)) AND PUBYEAR > 2009 AND (LIMIT-TO (DOCTYPE, “ar”) OR LIMIT-TO (DOCTYPE, “ip”) OR LIMIT-TO (DOCTYPE, “sh”)) AND (LIMIT-TO (LANGUAGE, “English”) OR LIMIT-TO (LANGUAGE, “Spanish”))	938
S2	(KEY (“anxiety”) OR KEY (“anxious”)) AND PUBYEAR > 2009 AND (LIMIT-TO (DOCTYPE, “ar”)) AND (LIMIT-TO (LANGUAGE, “English”) OR LIMIT-TO (LANGUAGE, “Spanish”))	94,305
S3	(KEY (“depression”) OR KEY (“depressive”)) AND PUBYEAR > 2009 AND (LIMIT-TO (DOCTYPE, “ar”) OR LIMIT-TO (DOCTYPE, “ip”)) AND (LIMIT-TO (LANGUAGE, “English”) OR LIMIT-TO (LANGUAGE, “Spanish”))	153,803
S4	KEY (suicid*) AND PUBYEAR > 2009 AND (LIMIT-TO (DOCTYPE, “ar”) OR LIMIT-TO (DOCTYPE, “ip”)) AND (LIMIT-TO (LANGUAGE, “English”) OR LIMIT-TO (LANGUAGE, “Spanish”))	27,891
S5	((KEY (“parenting styles”) OR KEY (“parenting practices”) OR KEY (“parental socialization”)) AND PUBYEAR > 2009) AND (((KEY (“depression”) OR KEY (“depressive”)) AND PUBYEAR > 2009) OR (KEY (suicid*) AND PUBYEAR > 2009) OR ((KEY (“anxiety”) OR KEY (“anxious”)) AND PUBYEAR > 2009)) AND (LIMIT-TO (LANGUAGE, “English”) OR LIMIT-TO (LANGUAGE, “Spanish”)) AND (LIMIT-TO (DOCTYPE, “ar”) OR LIMIT-TO (DOCTYPE, “ip”) OR LIMIT-TO (DOCTYPE, “sh”))	97

**Table 3:** Search Strategy in Pubmed.

Search Number	Search Terms	Results
#1	Search ((parenting styles[MeSH Terms]) OR parenting practices[MeSH Terms]) OR parental socialization[MeSH Terms] Filters: Publication date from 2010/01/01 to 2019/12/31	395
#2	Search (anxiety[MeSH Terms]) OR anxious[MeSH Terms] Filters: Publication date from 2010/01/01 to 2019/12/31	33,551
#3	Search (depression[MeSH Terms]) OR depressive[MeSH Terms] Filters: Publication date from 2010/01/01 to 2019/12/31	81,780
#4	Search suicid*[MeSH Terms] Filters: Publication date from 2010/01/01 to 2019/12/31	18,960
#5	Search ((((((((anxiety[MeSH Terms]) OR anxious[MeSH Terms]) AND (“2010/01/01”[PDat]: “2019/12/31”[PDat]))) OR (((depression[MeSH Terms]) OR depressive[MeSH Terms]) AND (“2010/01/01”[PDat]: “2019/12/31”[PDat]))) OR (suicid*[MeSH Terms] AND (“2010/01/01”[PDat]: “2019/12/31”[PDat]))) AND (“2010/01/01”[PDat]: “2019/12/31”[PDat]))) AND ((((parenting styles[MeSH Terms]) OR parenting practices[MeSH Terms]) OR parental socialization[MeSH Terms]) AND (“2010/01/01”[PDat]: “2019/12/31”[PDat])) Filters: Publication date from 2010/01/01 to 2019/12/31	27

**Table 4:** Search Strategy in Web of Science.

Search Number	Search Terms	Results
#1	TS = “parenting styles” OR TS = “parenting practices” OR TS = “parental socialization”	3343
#2	TS = anxiety OR TS = anxious	149,668
#3	TS = depression OR TS = depressive	257,204
#4	TS = suicid *	44,034
#5	#2 OR #3 OR #4	345,342
#6	#1 AND #5	686

Limiters: Timespan: 2010–2019. Databases: WOS, MEDLINE, SCIELO. Refined by: Document types: (article) and Languages: (English or Spanish). Note: The asterisk (*) acts as a truncation symbol to detect various results composed of a single string of text.

**Table 5:** Characteristics of the included studies.

Study Reference	Country	Study Type/Design	Sample Characteristics	*N* Age	Percentage of females	Parenting assessment	Outcome Variable	Measure
Andrade et al., 2012 [42]	Mexico	Cross-sectional	Community sample	1934 11–17 years	48.6	Andrade and Betancourt PPS	Depression	CES-D
Antón et al., 2016 [43]	Spain	Cross-sectional	Teenagers with a clinical history	100 13–17 years	70	PSS	Depression	SCL-90-R
Bahamón et al., 2018 [31]	Colombia	Cross-sectional	Community sample	328 15–18 years	53.4	PPS	Suicidal ideation	Scale developed for this study
Balan et al., 2017 [44]	Romania	Cross-sectional	Community sample	1132 10–14 years	46	APQ	Depression	YSR
Bámaca-Colbert et al., 2018 [45]	USA	Cross-sectional	Community sample	279 14–16 years	52	PBM	Depression	CES-D
Brassell et al., 2016 [46]	USA	Cross-sectional	Community sample	205 13–17 years	37.1	MAPS	Depression	BPC
Bullock et al., 2108 [47]	China	Cross-sectional	Community sample	462 mean age 13.4	46.7	PPC	Depression	CDI
Burlaka et al., 2017 [48]	Ukraine	Cross-sectional	Community sample	251 9–16 years	53	APQ	Depression	YSR
Carless et al., 2015 [49]	Australia	Cross-sectional	School refusing	60 12–17 years	46.7	FAD-GF	Depression	CDI
			School attending	46 12–17 years	60.9		Anxiety	SCARED
Castañeda et al., 2012 [50]	Spain	Cross-sectional	Adolescents who had assaulted their parents and adolescents who had not	28 mean age 15.7	35.7	ESPA29	Suicidal ideation	MACI
Chen et al., 2016 [51]	China	Cross-sectional	Monozygotic and same sex dizygotic twins	2230 10–18 years	52	Scale adapted from the IYFP	Anxiety	STAI
Costigan & Koryzma, 2011 [52]	Canada	Cross-sectional	Chinese immigrants in Canada	96 10–14 years	55.4	Scale adapted from the IYFP/ Parenting scale developed for this study	Depression	CES-D
Cruz et al., 2013 [53]	Portugal	Cross-sectional	Community sample with self-destructive thoughts	268 11–21 years	57.5	EMBU	Suicidal ideation	2 items from the YSR for suicidal ideation
			Clinical sample with self-destructive behaviours	42 11–21 years	86			
Cruz et al., 2014 [54]	Portugal	Cross-sectional	Community sample	1266 11–21 years	53	EMBU	Suicidal ideation	2 items from the YSR for suicidal ideation
Daryanani et al., 2016 [55]	USA	Longitudinal	Community sample	385 mean age 12.8	52.7	CRPBI	Depression	CDI
							Anxiety	MASC
Donath et al., 2014 [36]	Germany	Cross-sectional	Community sample	71891 mean age 15.3	48.7	Parenting scale developed for this study	Suicidal ideation	Scale developed for this study (2 items)
Dotterer & James, 2018 [56]	USA	Cross-sectional	Racial and ethnic minority sample	129 11–14 years	58	CRPBI	Depression	CDI
Doyle et al., 2017 [57]	USA	Cross-sectional	Community sample	660 11–21 years	47.4	PAM	Depression	CES-D
Eckshtain et al., 2010 [58]	USA	Cross-sectional	Diagnosis of type 1 or type 2 diabetes	61 10–18 years	62	APQ	Depression	CBCL
Eun et al., 2018 [59]	USA	Cross-sectional	Community sample	6483 13–18 years	-	PBI	Depression	CIDI
							Anxiety	
Flessner et al., 2017 [60]	USA	Cross-sectional	Anxious adolescents residing in location-based family public housing	81 9–17 years	61.7	PAKRS-PR // APQ	Anxiety	CBCL
Florenzano et al., 2011 [61]	Chile	Cross-sectional	Community sample	2346 13–20 years	59	CNAP	Suicidal ideation	Scale developed for this study (1 item)
Gagné & Melançon, 2013 [62]	Canada	Cross-sectional	Community sample	278 12–17 years	45.3	PVPPI	Depression	YSR
Gómez-Ortiz et al., 2016 [63]	Spain	Cross-sectional	Community sample	2060 12–19 years	48.0	PSS	Anxiety	SAS-A
Han & Grogan-Kaylor, 2013 [64]	Korea	Longitudinal	Community sample	3263 15–16 years	47.4	Scale adapted from the KYPS	Depression	Scale adapted from the KYPS
Jelenova et al., 2016 [65]	Czech Republic	Cross-sectional	Suffering inflammatory bowel disease	27 13–16 years	48.1	ADOR	Depression	CDI
Kerr et al., 2012 [66]	Sweden	Longitudinal	Community sample	1247 10–18 years	49.0	Parenting scale developed for this study	Depression	CES-D
Kim et al., 2013 [67]	USA	Longitudinal	Families in which both parents are foreign-born (Chinese)	379 12–15 years	54.4	Scale adapted from the IYFP	Depression	CES-D
King et al., 2016 [68]	USA	Cross-sectional	Data from the National Survey on Drug Use and Health (NSDUH)	17399 12–17 years	49.5	Interview	Depression	Interview
Lamis et al., 2012 [69]	USA	Longitudinal	High-risk schools	754 5–18 years	-	PDS	Depression	DISC
Liem et al., 2010 [70]	USA	Longitudinal	Community sample	1325 16–25 years	51.5	Authoritative parenting style	Depression	CES-D
Lipps et al., 2012 [71]	Caribbean islands: Jamaica, Bahamas, St. Kitts and Nevis, St. Vincent	Cross-sectional	Community sample	1955 13–18 years	52.1	Parenting Practices Scale	Depression	BDI
Logan et al., 2011 [72]	USA	Cross-sectional	High-risk pre/early adolescents	2598 12–15 years	50.6	Parenting scale developed for this study	Suicide ideation	Scale developed for this study (1 item)
Luebbe et al., 2018 [73]	China	Cross-sectional	Community sample	247 14–18 years	57.5	PCS	Anxiety	RCADS
Luyckx et al., 2011 [74]	Belgium	Cross-sectional	Congenital heart disease	429 14–18 years	46.6	Parental Regulation Scale & Psychological Control Scale from the YSR/Responsiveness scale from the CRPBI	Depression	CES-D
			Healthy controls	403 14–18 years	49.1			
Mak & Iacovou, 2018 [75]	USA	Longitudinal	Community sample	2954 15.4 mean age	-	Data from the Add Health study	Depression	CES-D
Meldrum et al., 2015 [76]	USA	Cross-sectional	Community sample	825 15 mean age	-	Parenting scale developed for this study	Depression	CDI
Moberg et al., 2011 [77]	Sweden	Longitudinal	Community sample: twins	2369 16–17 years	52.3	Expressed Emotion measure	Internalizing behaviour	CBCL YSR ABCL ASR
Mousavi et al., 2016 [78]	Malaysia	Cross-sectional	Community sample	227 13–18 years	46.3	EMBU	Anxiety	SCAS
Niditch & Varela, 2012 [79]	USA	Cross-sectional	Community sample	124 12–18 years	63	EMBU	Anxiety	RCMAS
Nunes & Mota, 2017 [80]	Portugal	Cross-sectional	Community sample	604 15–18 years	54.6	PSDQ-S	Suicidal ideation	SIQ
Ozer et al., 2013 [81]	USA	Longitudinal	Community sample Mexican American	151 12–15 years	45	PBF	Depression	BDI
Peng et al., 2011 [82]	China	Cross-sectional	Community sample	1083 13–18 years	51	Parenting scale developed for this study	Anxiety	SIAS
Pérez-Quiroz et al., 2013 [83]	Mexico	Cross-sectional	Community sample	393 15–17 years	56	Andrade and Betancourt PPS	Suicidal ideation	Scale developed for this study
Piko & Balázs, 2012 [84]	Hungary	Cross-sectional	Community sample	2072 12–21 years	50.8	Authoritative Parenting Index	Depression	CDI
Ruvalcaba et al., 2016 [85]	Mexico	Cross-sectional	Community sample	417 12–16 years	56	Andrade and Betancourt PPS	Depression	CDI
							Anxiety	RCMAS
Sanjeevan & de Zoysa, 2018 [86]	Sri Lanka	Cross-sectional	Community sample	232 15–18 years	53.9	Scale of Parenting Styles	Depression	DASS-21
							Anxiety	
Scharf et al., 2016 [87]	Israel	Cross-sectional	Community sample	3496	53.9	WPI/PCS/RMFQ/PAQ/PMQ	Depression	YSR
Sharaf et al., 2016 [88]	Egypt	Cross-sectional	Hospitalized for suicide attempt	150 13–21 years	92.7	PBI	Depression	CES-D
							Suicidal ideation	SIS
Shishido & Latzman, 2017 [89]	USA	Cross-sectional	Community sample	174 10–16 years	0	APQ	Depression	YSR
Simons et al., 2013 [90]	USA	Longitudinal	Community sample: African American	889 mean age 12.5	53.8	Scale adapted from the IYFP	Depression	DISC
				767 mean age 15				
Tahmouresi et al., 2017 [91]	Iran	Cross-sectional	Community sample	103 11–14 years	-	PS	Depression	CDI
	Germany			118 11–14 years				
Tak et al., 2015 [92]	The Netherlands	Longitudinal		417 mean age 13.9	48	IM-P	Depression	CDI
Taylor et al., 2012 [93]	USA	Cross-sectional	Economically disadvantaged, African American	200 mean age 14.5	52	CRPBI	Anxiety	RBPC
Van Oort et al., 2011 [94]	The Netherlands	Longitudinal	Community sample: Data from the TRacking Adolescents’ Individual Lives Survey (TRAILS).	2230 10–12 years	51	EMBU	Anxiety	RCADS
				2149 12–15 years	51			
				1653 14–18 years	53			
Wang et al., 2015 [95]	Taiwan	Longitudinal	Community sample	1990 mean age 13.3	50.3	Parenting Practices	Depression	SCL-90-R Short form
Wang et al., 2016 [96]	China	Cross-sectional	Community sample	589 12–19 years	57	PBM	Depression	Scale developed for this study
Xu et al., 2017 [97]	China	Cross-sectional	Migrant families from non-government-funded schools	1345 11–19 years	40.7	EMBU	Anxiety	Social Anxiety Subscale of SCS
Zhang et al., 2016 [98]	China	Cross-sectional	Community sample	3957 11–20 years	53.5	EMBU	Depression	SCL-90-R
							Anxiety	
							Suicidal ideation	Scale developed for this study

Abbreviations: ABCL: Adult Behaviour Checklist; APQ: Alabama Parenting Questionnaire; ASR: Adult Self Report; BPC: Brief Problem Checklist; CDI: Children’s Depression Inventory; CES-D: Center for Epidemiologic Studies Depression Scale; CIDI: Composite International Diagnostic Interview; CNAP: Cross National Adolescent Program; CRPBI: Child’s Report of Parental Behaviour Inventory; DISC: Diagnostic Interview Schedule for Children; EMBU: Inventory for Assessing Memories of Parental Rearing Behaviour; ESPA29: Escala de Socialización Parental en la Adolescencia [Parental Socialization in Adolescence Scale]; FAD-GF: General Functioning subscale of the Family Assessment Device; IM-P: Interpersonal Mindfulness in Parenting Scale; IYFP: Iowa Youth and Families Project; KYPS: Korea Youth Panel Survey; MACI: Millon Adolescent Clinical Inventory; MAPS: The Multidimensional Assessment of Parenting Scale; MASC: Multidimensional Anxiety Scale for Children; PAKRS-PR: Parenting Anxious Kids Ratings Scale-Parent Report; PAM: Parental Attitude Measure; PAQ: Parental Authority Questionnaire; PBF: Parent Behaviour Form; PBI: Parenting Bonding Instrument; PBM: Parent Behaviour Measure; PCS: Psychological Control Scale; PDS: Parental Discipline Scale; PMQ: Parental Monitoring Questionnaire; PPS: Parental Practices Scale; PS: Parenting Scale; PSDQ-S: Parenting Styles & Dimensions Questionnaire Short Version; PSS: Parental Socialization Style; PVPPI: Psychologically Violent Parental Practices Inventory; RBPC: Revised Behaviour Problem Checklist; RCADS: Revised Child Anxiety and Depression Scale; RCMAS: Revised Children’s Manifest Anxiety Scale; RMFQ: Relationship with Mother-Father Questionnaire; SAD: Scale of Anxiety in Children; SAS-A: Social Anxiety Scale for Adolescents; SCARED: Screen for Child Anxiety Related Emotional Disorders; SCAS: Spence Children’s Anxiety Scale; SCL-90-R: Symptom Checklist; SCS: Self-Consciousness Scale; SIAS: Social Interaction Anxiety Scale; SIQ: Suicidal Ideation Questionnaire; SIS: Suicide Intent Scale; STAI: State Trait Anxiety Inventory; WPI: Weinberger Parenting Inventory; YSR: Youth Self Report.

## References

[1] Bowlby J. (1969/1982). Attachment and Loss: Vol. 1. Attachment.

[2] Bowlby J. (1973). Attachment and Loss: Vol. 2. Separation: Anxiety and Anger.

[3] Bowlby J. (1980). Attachment and Loss: Vol. 3. Loss: Sadness and Depression.

[4] Bandura A., Walters R.H. (1963). Social Learning and Personality Development.

[5] Darling N., Steinberg L. (1993). Parenting style as context: An integrative model. Psychol. Bull..

[6] Baumrind D. (1966). Effects of authoritative parental control on child behavior. Child. Dev..

[7] Maccoby E.E., Martin J.A., Mussen P.H., Hetherington E.M. (1983). Socialization in the Context of the Family: Parent-Child Interaction. Handbook of Child Psychology.

[8] Lamborn S.D., Mounts N.S., Steinberg L., Dornbusch S.M. (1991). Patterns of competence and adjustment among adolescents from authoritative, authoritarian, indulgent and neglectful families. Child. Dev..

[9] Steinberg L., Lamborn S.D., Darling N., Mounts N.S., Dornbusch S.M. (1994). Over-time changes in adjustment and competence among adolescents from authoritative, authoritarian, indulgent, and neglectful families. Child. Dev..

[10] Akcinar B., Baydar N. (2014). Parental control is not unconditionally detrimental for externalizing behaviors in early childhood. Int. J. Behav. Dev..

[11] Barber B.K., Olsen J.E., Shagle S.C. (1994). Associations between parental psychological and behavioral control and youth internalized and externalized behaviors. Child. Dev..

[12] Pinquart M. (2017). Associations of parenting dimensions and styles with internalizing symptoms in children and adolescents: A meta-analysis. Marriage Fam. Rev..

[13] Barber B.K. (1996). Parental psychological control: Revisiting a neglected construct. Child. Dev..

[14] Barber B.K., Harmon E.L., Barber B.K. (2002). Violating the Self: Parental Psychological Control of Children and Adolescents. Intrusive Parenting: How Psychological Control affects Children and Adolescents.

[15] McLeod B.D., Wood J.J., Weisz J.R. (2007). Examining the association between parenting and childhood anxiety: A meta-analysis. Clin. Psychol. Rev..

[16] Yap M.B.H., Pilkington P.D., Ryan S.M., Jorm A.F. (2014). Parental factors associated with depression and anxiety in young people: A systematic review and meta-analysis. J. Affect. Disord..

[17] García O.F., Serra E., Zacares J.J., García F. (2018). Parenting styles and short- and long-term socialization outcomes: A study among Spanish adolescents and older adults. Psychosoc. Interv..

[18] Martínez I., García F., Musitu G., Yubero S. (2012). Family socialization practices: Factor confirmation of the Portuguese version of a scale for their measurement. Rev. Psicodidact..

[19] Musitu G., García J.F. (2001). Escala de Socialización Parental en la Adolescencia (ESPA29).

[20] Schaefer E.S. (1959). A circumplex model for maternal behavior. J. Abnorm. Child. Psychol..

[21] Delgado A.O., Jiménez Á.P., Sánchez-Queija I., Gaviño F.L. (2007). Maternal and paternal parenting styles: Assessment and relationship with adolescent adjustment. Anu. Psicol..

[22] García F., Gracia E., Selin H. (2014). The Indulgent Parenting Style and Developmental Outcomes in South European and Latin American Countries. Parenting Across Cultures: Childrearing, Motherhood and Fatherhood in non-Western Cultures.

[23] Martínez E., Julián A. (2017). Relación entre los estilos educativos parentales o prácticas de crianza y la ansiedad infanto-juvenil: Una revisión bibliográfica [The relationship between parenting styles or parenting practices, and anxiety in childhood and adolescence: A systematic review]. Rev. Esp. Pedagog..

[24] Yap M.B.H., Jorm A.F. (2015). Parental factors associated with childhood anxiety, depression, and internalizing problems: A systematic review and meta-analysis. J. Affect. Disord..

[25] Wood J.J., McLeod B.D., Sigman M., Hwang W.-C., Chu B.C. (2003). Parenting and childhood anxiety: Theory, empirical findings, and future directions. J. Child. Psychol. Psyc..

[26] Liu Y., Merritt D.H. (2018). Examining the association between parenting and childhood depression among Chinese children and adolescents: A systematic literature review. Child. Youth Serv. Rev..

[27] McLeod B.D., Weisz J.R., Wood J.J. (2007). Examining the association between parenting and childhood depression: A meta-analysis. Clin. Psychol. Rev..

[28] León-del-Barco B., Mendo-Lázaro S., Polo-del-Río M.I., López-Ramos V.M. (2019). Parental psychological control and emotional and behavioral disorders among Spanish adolescents. Int. J. Environ. Res. Public Health.

[29] Oliva A., Parra Á., Sánchez-Queija I., López F. (2007). Estilos educativos materno y paterno: Evaluación y relación con el ajuste adolescente [Maternal and paternal parenting styles: Assessment and relationship with adolescent adjustment]. An. Psicol..

[30] Pettit G.S., Laird R.D., Barber B.K. (2002). Psychological control and monitoring in early adolescence: The role of parental involvement and earlier child adjustment. Intrusive Parenting: How Psychological Control Affects Children and Adolescents.

[31] Bahamón M.J., Alarcón-Vásquez Y., Reyes L., Trejos A.M., Uribe J.I., García C. (2018). Prácticas parentales como predictoras de la ideación suicida en adolescentes colombianos [Parenting practices, predictor of Colombian adolescents suicidal ideation]. Psicogente.

[32] Cero I., Sifers S.K. (2013). Parenting behavior and the interpersonal-psychological theory of suicide: A mediated moderation analysis with adolescents. J. Affect. Disord..

[33] Lo H.H., Kwok S.Y., Yeung J.W., Low A.Y., Tam C.H. (2017). The moderating effects of gratitude on the association between perceived parenting styles and suicidal ideation. J. Child. Fam. Stud..

[34] Wong I.N., de Man A.F., Leung P.W.L. (2002). Perceived parental child rearing and suicidal ideation in Chinese adolescents. Soc. Behav. Personal..

[35] Konopka A., Rek-Owodziń K., Pełka-Wysiecka J., Samochowiec J. (2018). Parenting style in family and the risk of psychopathology. Postepy. Hig. Med. Dosw..

[36] Donath C., Graessel E., Baier D., Bleich S., Hillemacher T. (2014). Is parenting style a predictor of suicide attempts in a representative sample of adolescents?. BMC Pedriatr..

[37] King K.A., Vidourek R.A., Yockey R.A., Merianos A.L. (2018). Impact of parenting behaviors on adolescent suicide based on age of adolescent. J. Child. Fam. Stud..

[38] Martin G., Waite S. (1994). Parental bonding and vulnerability to adolescent suicide. Acta Psychiatr. Scand..

[39] Möller E.L., Nikolić M., Majdandžić M., Bögels S.M. (2016). Associations between maternal and paternal parenting behaviors, anxiety and its precursors in early childhood: A meta-analysis. Clin. Psychol. Rev..

[40] van der Bruggen C.O., Stams G.J.J.M., Bögels S.M. (2008). Research review: The relation between child and parent anxiety and parental control: A meta-analytic review. J. Child. Psychol. Psyc..

[41] Moher D., Liberati A., Tetzlaff J., Altman D.G. (2009). Guidelines and guidance preferred reporting items for systematic reviews and meta-analyses: The PRISMA statement. Ann. Intern. Med..

[42] Andrade P., Betancourt D., Vallejo A., Celis B.S., Rojas R.M. (2012). Parenting practices and depressive symptomatology in adolescents. Salud Ment..

[43] Antón J.M., Seguí D., Antón L., Barrera A. (2016). Relación entre estilos parentales, intensidad psicopatológica y tipo de sintomatología en una muestra clínica adolescente [Relationship between parenting styles, psychopathological intensity and type of symptoms in a adolescents clinical sample]. An. Psicol..

[44] Balan R., Dobrean A., Roman G.D., Balazsi R. (2017). Indirect effects of parenting practices on internalizing problems among adolescents: The role of expressive suppression. J. Child. Fam. Stud..

[45] Bámaca-Colbert M.Y., Gonzales-Backen M., Henry C.S., Kim P.S.Y., Roblyer M.Z., Plunkett S.W., Sands T. (2018). Family profiles of cohesion and parenting practices and Latino youth adjustment. Fam. Proc..

[46] Brassell A.A., Rosenberg E., Parent J., Rough J.N., Fondacaro K., Seehuus M. (2016). Parent’s psychological flexibility: Associations with parenting and child psychosocial well-being. J. Contextual Behav. Sci..

[47] Bullock A., Liu J., Cheah C.S.L., Coplan R.J., Chen X., Li D. (2018). The role of adolescents’ perceived parental psychological control in the links between shyness and socio-emotional adjustment among youth. J. Adolesc..

[48] Burlaka V., Kim Y.J., Crutchfield J.M., Lefmann T.A. (2017). Predictors of internalizing behaviors in Ukrainian children. Fam. Relat..

[49] Carless B., Melvin G.A., Tonge B.J., Newman L.K. (2015). The role of parental self-efficacy in adolescent school-refusal. J. Fam. Psychol..

[50] Castañeda A., Garrido-Fernández M., Lanzarote M.D. (2012). Menores con conducta de maltrato hacia los progenitores: Un estudio de personalidad y estilos de socialización [Juvenile offenders who assault their parents: A study of personality traits and parenting styles]. Rev. Psicol. Soc..

[51] Chen J., Yu J., Zhang J. (2016). Investigating unique environmental influences of parenting practices on youth anxiety: A monozygotic twin differences study. Int. J. Behav. Dev..

[52] Costigan C.L., Koryzma C.M. (2011). Acculturation and adjustment among immigrant Chinese parents: Mediating role of parenting efficacy. J. Couns. Psychol..

[53] Cruz D., Narciso I., Muñoz M., Pereira C.R., Sampaio D. (2013). Adolescents and self-destructive behaviours: An exploratory analysis of family and individual correlates. Behav. Psychol..

[54] Cruz D., Narciso I., Pereira C.R., Sampaio D. (2014). Risk trajectories of self-destructiveness in adolescence: Family core influences. J. Child. Fam. Stud..

[55] Daryanani I., Hamilton J.L., Abramson L.Y., Alloy L.B. (2016). Single mother parenting and adolescent psychopathology. J. Abnorm. Child. Psychol..

[56] Dotterer A.M., James A. (2018). Can parenting microprotections buffer against adolescents’ experiences of racial discrimination?. J. Youth Adolesc..

[57] Doyle O., Goings T.C., Cryer-Coupet Q.R., Lombe M., Stephens J., Nebbitt V.E. (2017). Paternal caregivers’ parenting practices and psychological functioning among African American youth living in urban public housing. Fam. Proc..

[58] Eckshtain D., Ellis D.A., Kolmodin K., Naar-King S. (2010). The effects of parental depression and parenting practices on depressive symptoms and metabolic control in urban youth with insulin dependent diabetes. J. Pediatr. Psychol..

[59] Eun J.D., Paksarian D., He J.-P., Merikangas K.R. (2018). Parenting style and mental disorders in a nationally representative sample of US adolescents. Soc. Psychiatry Psychiatr. Epidemiol..

[60] Flessner C.A., Murphy Y.E., Brennan E., D’Auria A. (2017). The Parenting Anxious Kids Ratings Scale-Parent Report (PAKRS-PR): Initial scale development and psychometric properties. Child. Psychiatry Hum. Dev..

[61] Florenzano R., Valdés M., Cáceres E., Santander S., Aspillaga C., Musalem C. (2011). Relación entre ideación suicida y estilos parentales en un grupo de adolescentes chilenos [Relation between suicidal ideation and parenting styles among a group of Chilean adolescents]. Rev. Med. Chile.

[62] Gagné M.H., Melançon C. (2013). Parental psychological violence and behavioral adjustment: The role of coping and social support. J. Interpers. Violence.

[63] Gómez-Ortiz O., Casas C., Ortega-Ruiz R. (2016). Ansiedad social en la adolescencia: Factores psicoevolutivos y de contexto familiar [Social anxiety in adolescence: Psycho- evolutionary and family factors]. Behav. Psychol..

[64] Han Y., Grogan-Kaylor A. (2013). Parenting and youth psychosocial well-being in South Korea using fixed-effects models. J. Fam. Issues.

[65] Jelenova D., Prasko J., Ociskova M., Latalova K., Karaskova E., Hruby R., Kamaradova D., Mihal V. (2016). Quality of life and parental styles assessed by adolescents suffering from inflammatory bowel diseases and their parents. Neuropsych. Dis. Treat..

[66] Kerr M., Stattin H., Özdemir M. (2012). Perceived parenting style and adolescent adjustment: Revisiting directions of effects and the role of parental knowledge. Dev. Psychol..

[67] Kim S.Y., Chen Q., Wang Y., Shen Y., Orozco-Lapray D. (2013). Longitudinal linkages among parent-child acculturation discrepancy, parenting, parent-child sense of alienation, and adolescent adjustment in Chinese immigrant families. Dev. Psychol..

[68] King K.A., Vidourek R.A., Merianos A.L. (2016). Authoritarian parenting and youth depression: Results from a national study. J. Prev. Interv. Community.

[69] Lamis D.A., Malone P.S., Lansford J.E., Lochman J.E. (2012). Maternal depressive symptoms as a predictor of alcohol use onset and heavy episodic drinking in youth. J. Consult. Clin. Psychol..

[70] Liem J.H., Cohen E., Lustig K. (2010). The influence of authoritative parenting during adolescence on depressive symptoms in young adulthood: Examining the mediating roles of self-development and peer support. J. Genet. Psychol..

[71] Lipps G., Lowe G.A., Gibson R.C., Halliday S., Morris A., Clarke N., Wilson R.N. (2012). Parenting and depressive symptoms among adolescents in four Caribbean societies. Child. Adolesc. Psychiatry Ment. Health.

[72] Logan J.E., Crosby A.E., Hamburger M.E. (2011). Suicidal ideation, friendships with delinquents, social and parental connectedness, and differential associations by sex: Findings among high-risk pre/early adolescent population. Crisis.

[73] Luebbe A.M., Tu C., Fredrick J.W. (2018). Socialization goals, parental psychological control, and youth anxiety in Chinese students: Moderated indirect effects based on school type. J. Youth Adolesc..

[74] Luyckx K., Goossens E., Missotten L., Moons P. (2011). Adolescents with congenital heart disease: The importance of perceived parenting for psychosocial and health outcomes. J. Dev. Behav. Pediatr..

[75] Mak H.W., Iacovou M. (2018). Dimensions of the parent-child relationship: Effects on substance use in adolescence and adulthood. Subst. Use Misuse.

[76] Meldrum R.C., Barnes J.C., Hay C. (2015). Sleep deprivation, low self-control, and delinquency: A test of the strength model of self-control. J. Youth Adolesc..

[77] Moberg T., Lichtenstein P., Forsman M., Larsson H. (2011). Internalizing behavior in adolescent girls affects parental emotional overinvolvement: A cross-lagged twin study. Behav. Genet..

[78] Mousavi S.E., Low W.Y., Hashim A.H. (2016). Perceived parenting styles and cultural influences in adolescent’s anxiety: A cross-cultural comparison. J. Child. Fam. Stud..

[79] Niditch L.A., Varela R.E. (2012). Perceptions of parenting, emotional self-efficacy, and anxiety in youth: Test of a mediational model. Child. Youth Care Forum.

[80] Nunes F., Mota C.P. (2017). Parenting styles and suicidal ideation in adolescents: Mediating effect of attachment. J. Child. Fam. Stud..

[81] Ozer E.J., Flores E., Tschann J.M., Pasch L.A. (2013). Parenting style, depressive symptoms, and substance use in Mexican American adolescents. Youth Soc..

[82] Peng Z.-W., Lam L.T., Jin J. (2011). Factors associated with social interaction anxiety among Chinese adolescents. East. Asian Arch. Psychiatry.

[83] Pérez-Quiroz A., Uribe J.I., Alexandra M., Bahamón M.J., Verdugo J.C., Ochoa S. (2013). Estilos parentales como predictores de ideación suicida en estudiantes adolescentes [Parenting styles as predictors of suicidal ideation in adolescents]. Psicol. Caribe.

[84] Piko B.F., Balázs M.Á. (2012). Control or involvement? Relationship between authoritative parenting style and adolescent depressive symptomatology. Eur. Child. Adolesc. Psychiatry.

[85] Ruvalcaba N.A., Gallegos J., Caballo V.E., Villegas D. (2016). Prácticas parentales e indicadores de salud mental en adolescentes [Parenting practices and markers of mental health in adolescence]. Psicol. Caribe.

[86] Sanjeevan D., de Zoysa P. (2018). The association of parenting style on depression, anxiety and stress among Tamil speaking adolescents in the Colombo city. Sri Lankaj. Child. Health.

[87] Scharf M., Mayseless O., Rousseau S. (2016). When somatization is not the only thing you suffer from: Examining comorbid syndromes using latent profile analysis, parenting practices and adolescent functioning. Psychiat. Res..

[88] Sharaf A.Y., Thompson E.A., Abd El-Salam H.F. (2016). Perception of parental bonds and suicide intent among Egyptian adolescents. J. Child. Adolesc. Psychiatr. Nurs..

[89] Shishido Y., Latzman R.D. (2017). Mother-son discrepant reporting on parenting practices: The contribution of temperament and depression. J. Fam. Psychol..

[90] Simons L.G., Simons R.L., Su X. (2013). Consequences of Corporal Punishment Among African Americans: The Importance of Context and Outcome. J. Youth Adolesc..

[91] Tahmouresi N., Schmitz J., Bender C., Tuschen-Caffier B. (2017). The impact of culture on parenting and psychopathology in children: A comparative study between Iran and Germany. Iran. J. Psychiatry Behav. Sci..

[92] Tak Y.R., van Zundert R.M.P., Kleinjan M., Engels R.C.M.E. (2015). Mindful parenting and adolescent depressive symptoms: The few associations are moderated by adolescent gender and parental depressive symptoms. Mindfulness.

[93] Taylor R.D., Lopez E.I., Budescu M., McGill R.K. (2012). Parenting practices and adolescent internalizing and externalizing problems: Moderating effects of socially demanding kin relations. J. Child. Fam. Stud..

[94] Van Oort F.V.A., Greaves-Lord K., Ormel J., Verhulst F.C., Huizink A.C. (2011). Risk indicators of anxiety throughout adolescence: The TRAILS study. Depress. Anxiety.

[95] Wang Y.-C.L., Chan H.-Y., Lin C.-W., Li J.-R. (2015). Association of parental warmth and harsh discipline with developmental trajectories of depressive symptoms among adolescents in Chinese society. J. Fam. Psychol..

[96] Wang C., Xia Y., Li W., Wilson S.M., Bush K., Peterson G. (2016). Parenting behaviors, adolescent depressive symptoms, and problem behavior: The role of self-esteem and school adjustment difficulties among Chinese adolescents. J. Fam. Issues.

[97] Xu J., Ni S., Ran M., Zhang C. (2017). The relationship between parenting styles and adolescents’ social anxiety in migrant families: A study in Guangdong, China. Front. Psychol..

[98] Zhang J., Song J., Wang J. (2016). Adolescent self-harm and risk factors. Asia-Pac. Psychiat..

[99] Perris C., Jacobsson L., Lindström H., von Knorring L., Perris H. (1980). Development of a new inventory for assessing memories of parental rearing behaviour. Acta Psychiatr. Scand..

[100] Frick P.J. (1991). The Alabama Parenting Questionnaire (Unpublished Rating Scale).

[101] Frick P.J., Christian R.E., Wootton J.M. (1999). Age trends in the association between parenting practices and conduct problems. Behav. Modif..

[102] Schaefer E.S. (1965). Children’s reports of parental behavior: An inventory. Child. Dev..

[103] Andrade P.P., Betancourt O.D., Rivera A.S., Díaz-Loving R., Sánchez A.R., Reyes L.I. (2008). Prácticas Parentales: Una medición Integral. La Psicología Social en México XII.

[104] Parker G., Tupling H., Brown L.B. (1979). A parental bonding instrument. Brit. J. Med. Psychol..

[105] Bush K.R., Peterson G.W., Cobas J.A. (2002). Adolescent’s perceptions of parental behaviors as predictors of adolescent self-esteem in mainland China. Sociol. Inq..

[106] Lempers J.D., Clark-Lempers D., Simons R.L. (1989). Economic hardship, parenting, and distress in adolescence. Child. Dev..

[107] Kovacs M. (1992). The Children’s Depression Inventory Manual.

[108] Radloff L.S. (1977). The CES-D Scale: A self-report depression scale for research in the general population. Appl. Psych. Meas..

[109] Achenbach T.M., Rescorla L.A. (2001). Manual for the ASEBA School-Age Forms & Profiles.

[110] Derogatis L.R., Rickels K., Rock A.F. (1976). The SCL-90 and the MMPI: A step in the validation of a new self-report scale. Br. J. Psychiatry..

[111] Costello E.J., Edelbrock C.S., Costello A.J. (1985). Validity of the NIMH Diagnostic Interview Schedule for Children: A comparison between psychiatric and pediatric referrals. J. Abnorm. Child. Psychol..

[112] Beck A., Steer R. (1987). Beck Depression Inventory Manual.

[113] Chorpita B.F., Yim L., Moffitt C., Umemoto L.A., Francis S.E. (2000). Assessment of symptoms of DSM-IV anxiety and depression in children: A revised child anxiety and depression scale. Behav. Res. Ther..

[114] Reynolds C.R., Richmond B.O. (1978). What I think and feel: A revised measure of children’s manifest anxiety. J. Abnorm. Child. Psych..

[115] Reynolds W.M. (1988). Psychometric characteristics of the adult suicidal ideation questionnaire in college students. J. Pers. Assess..

[116] Beck A.T., Schuyler D., Herman I., Beck A.T., Resnik H.L.P., Lettieri D.J. (1974). Development of suicide intent scale. The Prediction of Suicide.

[117] Achenbach T.M. (1991). Manual for the Youth Self Report and 1991 Profile.

[118] Wagner B.M. (2009). Suicidal Behavior in Children and Adolescents.

[119] Herba C.M., Ferdinand R.F., van der Ende J., Verhulst F.C. (2007). Long-term associations of childhood suicide ideation. J. Am. Acad. Child. Adolesc. Psychiatry.

[120] García F., Gracia E. (2009). Is always authoritative the optimum parenting style? Evidence from Spanish families. Adolescence.

[121] García F., Serra E., García O.F., Martinez I., Cruise E. (2019). A third emerging stage for the current digital society? Optimal parenting styles in Spain, the USA, Germany, and Brazil. Int. J. Environ. Res. Public Health.

[122] Fuentes M.C., García F., Gracia E., Alarcón A. (2015). Parental socialization styles and psychological adjustment. A study in Spanish adolescents. Rev. Psicodidact..

